# Connection, confluence and convergence: a protocol for reviewing policies on antimicrobial resistance and plastic pollution

**DOI:** 10.1136/bmjopen-2025-108062

**Published:** 2026-04-09

**Authors:** Rupal Shah-Rohlfs, Jeniffer Landicho, Vivienne Endoma, Bianca Joyce Sornillo, Marina Treskova, Joacim Rocklöv, Shannon A McMahon, Mark Donald C Reñosa

**Affiliations:** 1 Heidelberg Institute of Global Health, University Hospital and Faculty of Medicine, University of Heidelberg, Heidelberg, Germany; 2 Department of Global Health, Hans Rosling Center for Population Health, University of Washington, Seattle, Washington, USA; 3 Department of Epidemiology and Biostatistics, Research Institute for Tropical Medicine – Department of Health, Manila, Philippines; 4 Department of Epidemiology and Global Health, Umeå University, Umea, Sweden; 5 Heidelberg Institute of Global Health and Interdisciplinary Centre for Scientific Computing, University of Heidelberg, Heidelberg, Germany; 6 International Health Department, Johns Hopkins Bloomberg School of Public Health, Baltimore, Maryland, USA

**Keywords:** Health policy, QUALITATIVE RESEARCH, Climate Change, PUBLIC HEALTH

## Abstract

**Abstract:**

**Introduction:**

Antimicrobial resistance (AMR) and plastic pollution are converging global crises that threaten both human health and environmental sustainability. Despite the growing recognition of these challenges, few legislative and policy frameworks acknowledge the complex interplay between antibiotic misuse and environmental plastic contamination. This protocol seeks to bridge that gap by critically examining policies in Europe and the Philippines, focusing on those that target antibiotic misuse and plastic pollution in human and animal health.

**Methods and analysis:**

Document analysis will be employed to systematically review relevant legislative and policy frameworks. We will retrieve laws, regulations and policy documents from official databases, government websites and other sources using broad inclusion criteria. The extraction process and analysis will be guided by the READ (Ready, Extract, Analyse, Distill) approach which will ensure a thorough examination of how these documents address the dual challenges of AMR and plastic pollution. Particular attention will be paid to identifying policy gaps, overlaps and synergies that may affect the overall effectiveness and coherence of current governmental responses.

**Ethics and dissemination:**

This policy review has been granted exemption from ethical review by the Research Institute for Tropical Medicine (RITM-IRB No. 2024-35), Philippines. The results are expected to provide a robust evidence base to inform the development of integrated policies at the nexus of global public health and environmental sustainability. Findings will be disseminated at academic conferences and peer-reviewed publications and to key stakeholders within European, Philippine, and international organisations.

**Trial registration number:**

The detailed protocol is pre-registered and openly available on the Open Science Framework (https://osf.io/3tkn2/overview).

**STRENGTHS AND LIMITATIONS OF THIS STUDY**

- The study will employ a policy document analysis using the READ (Ready, Extract, Analyse, Distill) approach alongside qualitative content analysis to provide a systematic, transparent and replicable approach.
- Data retrieval and extraction will follow scoping and systematic review best practices, with predefined inclusion and exclusion criteria applied across multiple legal and policy documents.
- The study design incorporates independent screening, double-coding and consensus discussions among multiple reviewers to enhance analytical rigour and interpretation.
- A comparison between Europe and the Philippines will allow methodological insights across two distinct governance contexts, although findings may not be generalisable to all settings.
- Some relevant materials may be excluded due to language/access limitations or the rapidly evolving nature of policy frameworks on antimicrobial resistance and plastic pollution.

## Introduction

 Antimicrobial resistance (AMR), plastic pollution and climate change are among the most pressing global public health challenges of our time.^1^ They are heavily interconnected, compounding their effects on human and animal health. Recent evidence reveals significant links between AMR and plastic pollution, particularly in environmental and aquatic ecosystems, where microplastics provide habitats for antibiotic-resistant micro-organisms, accelerating the acquisition and spread of AMR.^2^,^6^ Addressing these challenges is not only critical for public health but also essential for achieving the United Nations Sustainable Development Goal 14, which aims to protect marine ecosystems from pollution and other threats.^7^ Despite projections of increasing plastic pollution, significant knowledge gaps and uncertainties remain.^8 9^

The scale of plastic pollution is alarming, with estimates indicating a rapid acceleration in the accumulation of plastic waste.^10^ Microplastics, particles less than 5 mm in size resulting from the decomposition of larger plastic materials, have become omnipresent in terrestrial and aquatic ecosystems.^11 12^ Industrial operations, sewage discharge and human activities^13^ introduce particles into marine environments that defy removal by wastewater treatment facilities,^5 14^ fostering biofilms (plastisphere) that operate as breeding sites for antibiotic-resistance gene transfer and microbial growth.^2 5^ Given the durability of plastics, which persist for centuries, this highlights the urgent need to address plastic pollution in global AMR initiatives.^4 15 16^

AMR, a natural phenomenon intensified by human practices, emerges when bacteria, viruses, fungi and parasites develop mechanisms to withstand antimicrobial agents, including antibiotics, biocides and heavy metals.^17 18^ The World Health Organization (WHO) recognises AMR as a major public health concern,^18^ linking it to approximately 4.95 million deaths in 2019.^19^ AMR exacerbates infections, prolongs hospitalisations, illnesses and fatalities and places considerable economic burden on healthcare systems,^20^ making it a global health security threat.

The increasing threat of AMR in bacterial species is fuelled by the uncontrolled availability and use of antibiotics without prescription, a challenge observed in many parts of the world.^21^,^23^ The COVID-19 pandemic has further intensified the problem, disrupting antibiotic stewardship and contributing to increased use of antibiotics, personal protective equipment and biocides. This led to higher concentrations of antibiotics in wastewater treatment plants, which on release into the environment contribute to the contamination of aquatic ecosystems.^24 25^ In addition, the widespread use of antibiotics in agriculture, a practice observed in low- and middle-income countries (LMICs),^26^ has led to the accumulation of antibiotic residues in surface water, facilitating the development and spread of AMR.^27 28^ Faecal bacteria and animal waste residues further compound this issue.^29^

Climate change amplifies both AMR and plastic pollution challenges.^30 31^ Warming global conditions have been shown to accelerate bacterial reproduction rates and increase the prevalence of antibiotic resistance.^32^,^35^ Changes in climate can also intensify hospital-acquired infections and lead to higher antibiotic usage.^32^ Extreme weather events and natural disasters, such as flooding, overwhelm wastewater treatment systems, releasing untreated water loaded with AMR and plastic waste into the environment.^36 37^ These cascading effects are felt globally, with vulnerable populations in LMICs often facing greater challenges due to informal waste industries and marginalised communities exposed to the illegal waste trade, amplifying harm to ecosystems and human health.^38 39^

Thus, the interconnected threats of AMR and plastic pollution represent an urgent and escalating public health crisis. Environmental contamination from plastic waste provides surfaces for biofilms that harbour antibiotic-resistant bacteria, directly increasing the risk of life-threatening infections. Indirectly, these pollutants worsen mental health, elevate exposure to infectious diseases and impose heavy financial burdens on healthcare systems, particularly in vulnerable populations.^40^ Persistent exposure to these environmental stressors undermines health across the life course, necessitating comprehensive mitigation strategies.^38^

Efforts to mitigate AMR increasingly recognise the need for integrated approaches that extend beyond public health interventions, such as improving surveillance of antibiotic use and resistance, regulating over-the-counter antibiotic sales, promoting vaccine use and strengthening infection control and hygiene practices.^41^,^43^ The WHO has established the Tricycle protocol, a multisectoral framework for AMR surveillance on monitoring extended-spectrum β-lactamase-producing *Escherichia coli* across human, animal and environmental sectors.^44 45^ While effective, these strategies do not address the role of microplastics in facilitating the spread of AMR in the environment. Concurrently, many countries have initiated national action plans and roadmaps to tackle plastic pollution across the plastics lifecycle, particularly by curbing single-use plastics.^46^ However, the intersection between AMR and plastic pollution, within the nexus of climate change, remains largely absent from international and national policy frameworks.

International efforts to create a binding treaty on plastic pollution highlight the need to understand current regulatory frameworks.^47 48^ While existing research has examined policies on AMR and plastic pollution separately, few studies have analysed how legislation and policies address these interconnected challenges either directly or indirectly. Policy documents, shaped by complex social and political processes, reflect complex negotiations between governments, industry stakeholders and civil society organisations.^49 50^ Understanding these frameworks is essential for developing effective responses to the combined threats of AMR and plastic pollution.

While researchers have successfully conducted document analyses to examine complex environmental health challenges, including climate change adaptation in healthcare systems,^51^ and the integration of health considerations in national adaptation plans for AMR,^52^ we are not aware of research that unpacks policy responses at the nexus of AMR and plastic pollution. Previous document analyses have typically examined these issues separately, with studies of national action plans on AMR focusing primarily on health sector responses,^53^ while analyses of plastic pollution policies have largely concentrated on environmental regulations,^54^ with neither fully capturing the complex interactions between these domains.

To address these gaps, we will conduct a systematic policy review to map current legislation, identify necessary policy touchpoints and evaluate the alignment of initiatives targeting AMR and plastic pollution in the context of climate change. By examining environmental and water management frameworks in Europe and the Philippines, we aim to understand how different regulatory contexts approach policy integration for environmental AMR mitigation and plastic pollution. This study will analyse legal, policy and regulatory documents from Europe and the Philippines, focusing on health impacts and how these interconnected issues are recognised and acted on.

### Research aim and objectives

This research aims to establish a comprehensive knowledge base for policymakers in Europe and the Philippines, as part of the European Union (EU)-funded TULIP (community-based engagement and intervenTions to stem the spread of antimicrobial resistance in the aqUatic environments catalysed by cLImate change and Plastic pollution interactions) project, contributing to the project’s broader investigation of AMR-plastic pollution interactions in these regions, by examining existing legislation and policies addressing antibiotic misuse and plastic pollution. The study focuses on understanding how legal and policy instruments approach these challenges both separately and as interconnected issues affecting environmental and public health. By analysing these laws and policies across both regions, this research will identify opportunities for more integrated approaches that respond to the environmental pathways linking AMR and plastic pollution. Our search strategy is designed to identify relevant laws and policies across public health, environmental protection and agriculture sectors.

## Methods

### Study design

This study, a policy document analysis, compares how policies within Europe and the Philippines conceptualise and address AMR and plastic pollution. Policy document analysis is an essential and effective strategy in mapping existing frameworks and identifying explicit gaps in policies. Documents include but are not limited to international treaties, legal instruments (laws, regulations, acts, ordinances and executive orders), policy instruments (non-binding or strategy directives that guide implementation), including national adaptation plans, government statements, position papers, sector-level guidance, and traditional media and peer-reviewed publications.

**Figure 1:**
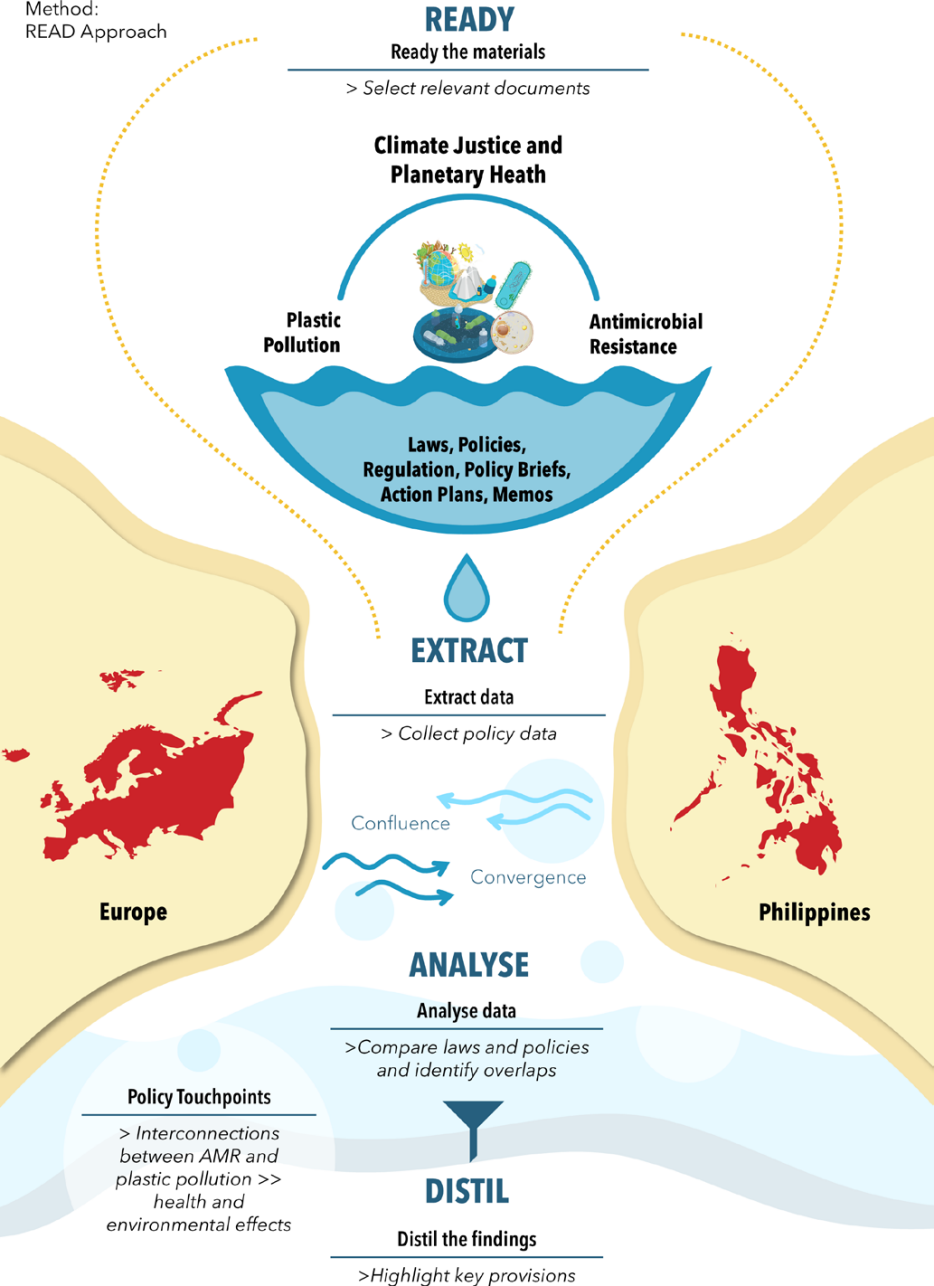
Applying the READ approach: a framework for policy analysis on plastic pollution and antimicrobial resistance in Europe and the Philippines. AMR, antimicrobial resistance; READ, Ready, Extract, Analyse, Distill.

Our overarching methodology is centred on the READ (Ready, Extract, Analyse, Distill) framework—a stepwise approach for rigorous document analysis—to guide data collection and analysis throughout the study.^55^ Our approach emphasises qualitative content analysis of policy texts while adhering to scoping and systematic review best practices for comprehensive search and transparent reporting.^56 57^ All protocol amendments will be tracked and updated on the Open Science Framework registration record and will be described in the resulting publications. An overview of the methodological workflow is presented in figure 1, which outlines the process from document identification to synthesis.

We will systematically synthesise European and Philippine governmental responses to AMR and plastic pollution by collecting and analysing primary legislative and policy instruments, with an explicit focus on reducing plastic pollution and addressing its connection to AMR. In alignment with the public policy process,^58^,^60^ we define AMR and plastic pollution laws and policies as government responses explicitly designed to reduce plastic leakage and combat the spread of AMR, beyond healthcare settings, focusing on environmental and community pathways rather than hospital-acquired infections. Given the significant leakage of plastic into the environment^12 61 62^ and the role of microplastics as potential vectors for antibiotic resistance genes in ecosystems,^63^ we will consider both targeted policies (addressing AMR and plastic pollution directly) and general waste management policies (addressing broader waste reduction and control) as part of the governmental response. Policies covering all stages of the plastic lifecycle, as well as those mitigating AMR transmission will be included to reflect their relevance to environmental and public health.

### Document analysis: the READ approach

The data extraction and analysis are guided by the READ approach as the core conceptual framework. READ is a systematic four-step method developed for qualitative health policy document analysis at both global and local levels.^55^ READ provides a clear structure for reviewing documents and ensuring analytical rigour.^55^ The key stages of the READ framework are illustrated in figure 1.

#### Ready materials: collection of legal and policy instruments

To identify the relevant sources for the policy review, we will conduct a comprehensive search using keywords or terms such as [“Antimicrobial Resistance” OR “AMR” OR “Plastic Pollution” OR “Plastics” OR “Plastic Garbage”] AND [“Policy” OR “Legal Instrument” OR “Strategies” OR “Plans”]. These search terms will help us capture publicly available national policies, legal instruments (including acts, resolutions and ordinances), strategic plans, guidelines and programme documents relevant to our aims. Our search will target official and institutional websites, policy databases (ie, Food and Agriculture Organisation, AMR-Lex, Codex Committee, Global Plastic Laws Database, Plastic Policy Inventory, LAW Phil Project), and PubMed, Scopus and Web of Science. We will also use Google Scholar and hand-pick other works cited or discussed in the policies included in the review as a secondary search of policy documents to identify grey literature and policies that may emerge during the data extraction.

In the Philippines, we will specifically search the official websites of government agencies involved in the One Health Programme and the Inter-Agency Committee on AMR. These agencies include the Departments of Health, Agriculture, Science and Technology, Trade and Industry, Environment and Natural Resources, and Interior and Local Government. Law and medical libraries in the Philippines will also be consulted to locate pertinent legal and policy documents. At the European level, we will review EU regulatory bodies, official EU databases and regional organisations engaged in AMR and plastic pollution. A steering committee within the EU-funded TULIP project will be regularly consulted to help with (1) clarifying profession-specific nomenclature and operational definitions around AMR and plastic pollution and (2) identifying key papers and grey literature.

For agencies or regions where initial searches yield no results, we will send targeted email requests to relevant bodies, including WHO Regional Offices, EU and Philippine agencies, and key contacts. If repeated outreach and follow-up searches reveal no relevant policies, the region or agency will be classified as having inaccessible policy availability.

To ensure that only relevant policy documents are included, we define inclusion and exclusion criteria a priori (see table 1). We will exclude documents that do not contain provisions that are relevant to the target issues, are not policy-oriented (eg, non-peer-reviewed research, personal opinion, unapproved documents), or are duplicative or superseded versions of laws and policies. Document searches began in August 2024; however, we will exclude materials that were published before 1990 unless they are of historical significance. It is anticipated that most documents identified will be in English. The review will primarily focus on English-language sources. When Filipino documents are located, they will be included and translated into English using machine translation tools (eg, Google Translate, DeepL). However, documents that cannot be retrieved in full or translated will be excluded to maintain the integrity and accessibility of the analysis. During the preparatory phase, we will establish a systematic filing and naming convention for ease of identification and document retrieval of included documents.

**Table 1:** Inclusion and exclusion criteria: laws, regulations and policies relevant to AMR and plastic pollution

Parameters	Inclusion criteria	Exclusion criteria
Topic	Laws, policies or legal framework on *AMR and plastic pollution*; documents addressing both topics or their intersection	Documents focused solely on other environmental issues or health topics; general pollution or resistance documents that make no specific reference to plastics or antimicrobials, unless they are indexed in AMR-specific repositories (eg, AMR-LEX) as addressing AMR concerns, regardless of whether plastics or antimicrobials are explicitly mentioned.
Sources of information	*International databases/inventories*: the Food and Agriculture Organisation (FAO), AMR-Lex (laws, regulations, and policies relevant to AMR), EUR-Lex (database of European Union Law and other public documents), Codex Committee, Global Plastic Laws Database, Plastics Policy Inventory Search, LAW Phil (a project of the Arellano Law Foundation in the Philippines to make available online, all Philippine legal materials), PubMed Scopus and Web of Science/Google Scholar *Official websites of government agencies*: participating in the One Health Programme and the Inter-agency Committee on Antimicrobial Resistance and the Ecological Solid Waste Management Act of 2000: Department of Health, Department of Agriculture, Department of Environment and Natural Resources, Department of Science and Technology, Department of Trade and Industry, Department of Interior and Local Government *Official websites of relevant European Union organisations*: European Commission, European Bioplastics, The European Parliament, The European Commission press corner, The Council of the European Union, European Environment Agency, European Chemicals Agency *Online searches*: Official Gazette, Google Search, WHO Library of AMR National Action Plans, World Bank, Eurostat *Libraries*: Law Libraries, Medical Libraries	Social media platforms Personal blogs Commercial websites with potential bias
Type of documents	*Official documents*: policies or policy directives, strategies for addressing the spread of plastic pollution and AMR, official statements and declarations, official position/white paper *Legal documents*: acts, laws, regulations, ordinances, executive orders, memorandum of understanding, cooperation agreements *Implementation documents*: standard operating procedures (SOPs)/training manuals *Working documents*: memoranda *Scholarly work*: peer-reviewed publications, including commentaries, viewpoints and editorials *Media and communications*: newspaper and magazine articles (reputable sources), podcasts, videos and radio and TV programmes, advertisements and posters, official Facebook pages of province, city and municipal government	Unpublished or non-peer-reviewed research Personal opinions or editorials (eg, newspapers, magazines, tabloid papers and unvetted social media platforms) Unofficial or unapproved documents Drafts or works in progress
Date of inclusion	All documents related to the plastic pollution and AMR will be included; however, if a document was superseded by a new version, we will include the latest version of the document	Outdated or superseded versions of documents; documents published before 1990 unless of historical significance
Language	Documents in English; documents in Filipino	Documents in languages other than those specified in the inclusion criteria
Geographical scope inclusion	National acts, policies and laws; international agreements and programmatic policy directions applicable to EU countries and the Philippines; local government policies if significant	Acts, policies or laws specific to other countries unless directly relevant to the EU and/or Philippine context

AMR, antimicrobial resistance; EU, European Union.

#### Extract the data

Once materials are gathered and categorised according to the inclusion and exclusion criteria, we will proceed with data extraction. As part of this process, no language restrictions will be applied. Acknowledging the broad definitions of ‘policy’ and ‘legal instrument’, we will include all national and agency documents outlining objectives, guidelines, legal actions or strategies related to AMR and/or plastic pollution. To ensure a comprehensive and inclusive analysis, no document will be excluded based solely on its title (eg, ‘act’, ‘resolution’ or ‘treaty’).

To extract the data, we will develop a structured data extraction form using Kobo Toolbox to systematically chart relevant information from each included document. Key data fields will include metadata (eg, document title, year, issuing authority, jurisdiction level) and content variables that are aligned with our review objectives—such as policy goals, target areas (human, animal and environment), described interventions, involved sectors and any noted links between AMR and plastic pollution. The extraction form will enable the four reviewers to work in parallel and enter data in a standardised way.

To optimise the review process and account for the distinct global discourses surrounding AMR and plastic pollution, researchers (RS-R and VE) will focus on documents related to plastic pollution, while researchers (JL and BJS) will concentrate on those pertaining to AMR. Each reviewer will independently screen the titles and abstracts and/or executive summaries (if available) of documents in their respective domains to determine relevance to the study objectives. A steering committee within TULIP will be regularly consulted to help with clarifying profession-specific nomenclature and operational definitions around AMR and plastic pollution. Documents that pass this initial screening will undergo a full-text review by the same reviewer to ensure they meet the inclusion criteria and do not fall under any exclusion criteria. In cases where a reviewer is uncertain about the inclusion of a document after full-text review, we will consult with the partner reviewer or the broader research team to make a final determination. Any disagreements that arise between the reviewers will be resolved through discussion or with the involvement of a third reviewer (MDR).

We will also extract the research evidence, guidelines or legal frameworks and other documents that were cited from the reviewed documents that are pertinent to the connection, confluence and convergence of AMR and plastic pollution, and enter them into Kobo Toolbox to ensure completeness of the data.

Data entered into Kobo Toolbox will be exported as an Excel spreadsheet for subsequent analysis. The structure follows a data extraction template detailed in the online supplemental file 1. As the review progresses, new categories and elements may be added to the data extraction tool and applied to the full dataset.

The exported Excel files will be regularly backed up to maintain an accurate record of the review process and facilitate data analysis. Periodic team meetings will be held to discuss progress, address challenges and ensure consistency in the review approach across both AMR and plastic pollution documents. Additionally, discussions will be facilitated through Zoom calls with the team and/or the steering committee to review emerging findings and enhance the rigour and trustworthiness of the data extraction process.

#### Analyse data

Extracted data will be analysed by examining both the similarities and differences within and between the identified laws and policies. The analysis will also focus on areas where policies overlap or intersect (policy touchpoints) concerning environmental, animal and human effects related to AMR and plastic pollution. We will categorise similar provisions and document key recommended practices through memos or reflexive notes, ensuring that connections and insights are captured for further discussion and interpretation.

Selected policy documents addressing plastic pollution and antibiotic misuse in humans and animals in Europe and the Philippines will be qualitatively coded line-by-line using NVivo 14 (QSR International, V.14, 2023) to identify and categorise the policy instruments used, the types of plastics and antibiotics targeted, and the stages of their respective lifecycles that are addressed. All four reviewers (RS-R, VE, JL and BJS) will independently review and code data using the Framework Analysis approach developed by Pope and colleagues,^64^ which will allow key themes to emerge from the policy content. The coding process will apply codes that characterise the suite of policy design options for each instrument, enabling us to distill these instruments into discrete categories and summarise overarching trends, including how these policies highlight connections and interactions between AMR and plastic pollution.^65^ Only the sections of the policies relevant to mitigating plastic pollution, antibiotic misuse and their intersection with AMR will be coded, allowing us to explore the convergence of these critical issues at the intersection of public health and environmental sustainability.

We aim to develop a summary of codes to establish a typology of policy instruments addressing AMR and plastic pollution, while identifying policy gaps and areas for improved integration. This typology will be derived from a review of legislative and policy frameworks in Europe and the Philippines. Given the anticipated complex nature of the legal and policy instruments we will review, the initial codebook may be challenging to apply. To address this, we plan to iteratively refine the codebook through coding sample policies from our inventory. After initial sampling, we will employ a simplified and more consistent typology aligned with the included policies. The codebook will be adapted during the analysis process, collaboratively finalised by the lead researchers and systematically applied across all policy documents.

The lead reviewers (RS-R, VE, JL and BJS) will compare notes and refine the coding scheme to ensure consistent interpretation of the documents. Codes will be organised into categories reflecting various aspects of policy responses (eg, regulatory approaches, public awareness initiatives, international collaboration, or One Health perspectives linking environmental and antimicrobial challenges). When coding discrepancies arise, the research team resolves them through discussion, with a third member available to arbitrate unresolved differences, thereby strengthening the reliability of the analysis.

#### Distill your findings

Following the analysis, we will review the memos to refine and complete any gaps for accuracy and consistency. We will synthesise the findings across all documents, distilling the identified themes into a narrative that answers our research question. Through this process, we will develop a theory of change to understand how plastic pollution and AMR policies interact. This will help assess the capacities needed for government agencies to effectively use and demand research evidence. We will use these insights to conduct a readiness assessment of relevant agencies and refine our integrated knowledge translation strategy accordingly.

### Current study status

The policy review commenced in August 2024 and is anticipated to conclude by 31 December 2026. The methodological approach was developed in collaboration with the broader team working on the TULIP project at the University of Heidelberg (Germany) and the Research Institute for Tropical Medicine (Philippines). Currently, documents are being retrieved (step 1 of the READ approach) and textual extraction has begun (step 2 of the READ approach).

### Patient and public involvement

There was no involvement from patients and/or the public in the design of this research, and no patients or members of the public will be involved in the conduct of the review. We will present the results of the review to stakeholders, policymakers and experts to get their feedback.

### Ethics and dissemination

This study has been reviewed and granted exemption from ethical review by the Research Institute for Tropical Medicine, Department of Health in the Philippines (RITM-IRB No. 2024-35). The exemption was granted on the grounds that, as determined by the reviewing Ethics Committee, the research involves no human participants, biological samples or personal data, and consists solely of a review of publicly available legislative and policy documents. A Certification of Exemption has been issued, and the research has been authorised to proceed.

Findings from this review will be disseminated through the EU-funded TULIP consortium. Results will also be shared with relevant national and international stakeholders and presented at academic conferences focused on global and public health, AMR, plastics and environmental governance. The study will result in peer-reviewed publications of at least two policy-focused manuscripts submitted to open-access international journals and will spearhead a scoping review.

## Discussion

This review uses the READ approach for document analysis in health policy research.^55^ This approach has been used to examine policy landscapes, as shown in studies of disaster governance, health systems and climate adaptation.^66^ For this review, the methodological approach was adapted in collaboration with the broader team working on the TULIP project at the University of Heidelberg, Germany, and the Research Institute for Tropical Medicine, Philippines, to identify policies, reforms and initiatives that affect efforts to reduce environmental AMR and plastic pollution.

We are applying the READ approach to study AMR and plastic pollution, identifying gaps and insights for policy development. Addressing this issue is critical, as current strategies often overlook connections like antimicrobial leakage into water systems and microplastics’ role in AMR spread. This review examines the need for policies that integrate environmental and health governance.

### Expanding the body of knowledge

In addition to identifying policy gaps, this review aims to expand the body of knowledge on the confluence of AMR and plastic pollution—an area that has received limited attention. These interconnected challenges are linked to planetary health concerns, including biodiversity loss, ecosystem degradation and public health inequities.^67^ By framing these intersections (policy touchpoints) as policy priorities, this review contributes to broader discussions on the need for integrated and holistic approaches to environmental and public health policymaking.

For example, antimicrobial leakage exacerbated by plastic pollution creates pathways for AMR to spread through aquatic ecosystems. However, few existing policies address these combined effects.^68^ By drawing attention to such overlooked areas, this review can catalyse further research and advocacy, underscoring the importance of cohesive responses that align with global sustainability goals. Although previous studies have focused on national action plans for both AMR and plastic pollution,^53 69^ there has been limited exploration of legal and regulatory instruments and their role in AMR and plastic governance, highlighting a research gap.

The findings of this review will also inform a systematic scoping review of policy-related research, culminating in a report with analysis, recommendations and an action plan for policymakers and decision-makers. This output will support the development of integrated and community-based interventions that are culturally adapted to both local challenges and global priorities.

### Congruence, confluence and epistemic injustices

This review also emphasises the importance of achieving congruence and confluence in policy frameworks, particularly in addressing epistemic injustices, the unequal flow and application of knowledge across different governance levels. Global policies are often not adequately translated into local contexts, and conversely, local innovations are frequently overlooked in global policy discussions.^70^ These disparities can hinder the reach, adoption, effectiveness and scaling up of evidence-based interventions, perpetuating inequities in environmental and health outcomes.^71^

For instance, in the Philippines, international frameworks for addressing plastic pollution have informed local policy initiatives, but gaps remain in their implementation due to limited resources and insufficient community engagement.^72^ Similarly, EU policies demonstrate progress in integrating environmental and health objectives, but ensuring alignment across member states remains a challenge.^73^ This review identifies opportunities to connect global and local policies for effective interventions.

### Far-reaching implications

While this review focuses on Europe and the Philippines, its findings have implications for global efforts to combat AMR and plastic pollution. The review may encourage research into these challenges and discussions on policy development. These approaches help address health challenges across borders. By applying the READ approach and engaging with policymakers, academics, programme implementers and community stakeholders, this review ensures that its findings are both scientifically robust and practically relevant.

### Limitations

While the misuse of antibiotics and the issue of microplastics are global concerns, our policy review is limited to documents from Europe and the Philippines, potentially overlooking relevant policies from other regions addressing the nexus between AMR and plastic pollution. This protocol acknowledges a potential language limitation. Because the review will likely capture predominantly English-language documents, materials published in other languages may not be identified, potentially excluding relevant perspectives. Access to certain policy documents might be restricted, potentially affecting the comprehensiveness of our analysis. Furthermore, given the rapidly evolving nature of AMR and plastic pollution research, some findings may become outdated relatively quickly. These limitations should be considered when interpreting the forthcoming results of our policy review.

## Supplementary material

10.1136/bmjopen-2025-108062 online supplemental file 1
