# Supplementary material for: Connection, confluence and convergence: a protocol for reviewing policies on antimicrobial resistance and plastic pollution
Source: BMJ Open. 2026 Apr 9;16(4):e108062. doi: 10.1136/bmjopen-2025-108062 (PMC13084955; doi:10.1136/bmjopen-2025-108062)
Supplement: online supplemental file 1 [file bmjopen-16-4-s001.pdf]

KoboToolbox

AMR Data Extraction Tool

FILE NAME

COUNTRY
- ◯ Philippines
- ◯ European Union

DOCUMENT INFORMATION

TYPE OF DOCUMENTS	TYPE OF DOCUMENTS	SPECIFIC TYPE OF DOCUMENTS
**TYPE OF DOCUMENTS**	◯ Legal Documents<br>◯ Media and Communications<br>◯ Implementation Documents<br>◯ Working Documents<br>◯ Scholarly Work<br>◯ Official Documents	

DOCUMENT TITLE

FIRST AUTHOR
Last name, First Name

ALL AUTHORS
Last Name, First Name Initial

DATE OF PUBLICATION/APPROVAL
yyyy-mm-dd

PUBLISHER/JOURNAL TITLE

SOURCE
i.e., interviewee, MoH website, Pubmed

AMR FOCUS
- ☐ Human
- ☐ Animal
- ☐ Environment

REGULATORY FRAMEWORKS FOR ANTIMICROBIALS IN HUMAN (Manufacturer, Healthcare and Pharmacies)

GOVERNMENT AGENCIES

PRODUCTION

MARKET AUTHORIZATION AND REGULATORY APPROVAL

SELECTION, PROCUREMENT AND SUPPLY

APPROPRIATE AND PRUDENT USE

DISPOSAL BY THE END USER

REGULATORY FRAMEWORKS FOR TERRESTRIAL AND AQUATIC ANIMALS

GOVERNMENT AGENCIES

PRODUCTION

MARKET AUTHORIZATION AND REGULATORY APPROVAL

SELECTION, PROCUREMENT AND SUPPLY

APPROPRIATE AND PRUDENT USE

DISPOSAL BY THE END USER

REGULATORY FRAMEWORKS TO PREVENT CONTAMINATION IN FOOD AND THE ENVIRONMENT

GOVERNMENT AGENCIES

FOCUS
- ☐ Human
- ☐ Animal

APPROPRIATE AND PRUDENT USE

DISPOSAL BY THE END USER

FOOD SAFETY

ENVIRONMENT WASTE

WATER

REGULATORY FRAMEWORKS FOR PLASTIC POLLUTION

GOVERNMENT AGENCIES

PRODUCTION

REUSE

END OF LIFE

OTHER INFORMATION

SUMMARY OF DOCUMENT TYPE AND OBJECTIVE

SUMMARY OF KEY DOCUMENT ANALYSIS FINDINGS
Include direct quotes & references to page numbers where appropriate

RESEARCH EVIDENCE OR OTHER WORKS CITED OR DISCUSSED

MENTION OF INTERNATIONAL RECOMMENDATIONS/DECISIONS

BUDGET/FINANCING INFORMATION

KEYWORDS

POTENTIAL RESPONDENTS NAME

TIMELINE EXTRACTED
yyyy-mm-dd hh:mm

FULL REFERENCE

URL

MATERIALS
- ◯ Hard Copy
- ◯ Digital Copy

REFLEXIVE NOTE

Save Draft | Submit

Powered by ENKETO
