# Supplementary material for: Connection, confluence and convergence: a protocol for reviewing policies on antimicrobial resistance and plastic pollution
Source: BMJ Open. 2026 Apr 9;16(4):e108062. doi: 10.1136/bmjopen-2025-108062 (PMC13084955; doi:10.1136/bmjopen-2025-108062)
Supplement: Supplementary file 2 [file bmjopen-2025-108062.reviewer_comments.pdf]

PEER REVIEW HISTORY

BMJ Open publishes all reviews undertaken for accepted manuscripts. Reviewers are asked to complete a checklist review form and are provided with free text boxes to elaborate on their assessment. These free text comments are reproduced below.

ARTICLE DETAILS

Title (Provisional)

Connection, Confluence, and Convergence: A Protocol for Reviewing Policies on Antimicrobial Resistance and Plastic Pollution

Authors

Shah-Rohlfs, Rupal; Landicho, Jeniffer; Endoma, Vivienne; Sornillo, Bianca; Treskova, Marina; Rocklov, Joacim; McMahon, Shannon A.; Reñosa, Mark Donald

---

VERSION 1 - REVIEW

**Reviewer**	**1**
**Name**	**Sutherland, Kim**
**Affiliation**	**New South Wales Agency for Clinical Innovation**
**Date**	**02-Nov-2025**
**COI**	**None**

An interesting study, well conceived and designed. I initially had questions about how the two sets of researchers would come together to study the nexus between AMR and plastic pollution - but am assured the method developed will address this. I look forward to reading the results.

VERSION 1 - AUTHOR RESPONSE

**Reviewer 1 Comments**	
An interesting study, well conceived and designed. I initially had questions about how the two sets of researchers would come together to study the nexus between AMR and plastic pollution – but am assured the method developed will address this. I look forward to reading the results.	We sincerely thank **Reviewer 1** for their positive and encouraging comments. We appreciate their recognition that the study is well conceived and designed. We are pleased that the revised description of our methodology has clarified how the two research teams will collaborate to address the intersection between AMR and plastic pollution. We look forward to sharing the outcomes of this work in due course.
